# Comparison of modelled diffusion-derived electrical conductivities found using magnetic resonance imaging

**DOI:** 10.3389/fradi.2025.1492479

**Published:** 2025-01-22

**Authors:** Sasha Hakhu, Leland S. Hu, Scott Beeman, Rosalind J. Sadleir

**Affiliations:** ^1^ School of Biological and Health Systems Engineering, Arizona State University, Tempe, AZ, United States; ^2^ Department of Radiology, Mayo Clinic Arizona, Phoenix, AZ, United States

**Keywords:** electrical conductivity, diffusion, magnetic resonance imaging, microstructure imaging, electrodeless methods

## Abstract

**Introduction:**

Magnetic resonance-based electrical conductivity imaging offers a promising new contrast mechanism to enhance disease diagnosis. Conductivity tensor imaging (CTI) combines data from MR diffusion microstructure imaging to reconstruct electrodeless low-frequency conductivity images. However, different microstructure imaging methods rely on varying diffusion models and parameters, leading to divergent tissue conductivity estimates. This study investigates the variability in conductivity predictions across different microstructure models and evaluates their alignment with experimental observations.

**Methods:**

We used publicly available diffusion databases from neurotypical adults to extract microstructure parameters for three diffusion-based brain models: Neurite Orientation Dispersion and Density Imaging (NODDI), Soma and Neurite Density Imaging (SANDI), and Spherical Mean technique (SMT) conductivity predictions were calculated for gray matter (GM) and white matter (WM) tissues using each model. Comparative analyses were performed to assess the range of predicted conductivities and the consistency between bilateral tissue conductivities for each method.

**Results:**

Significant variability in conductivity estimates was observed across the three models. Each method predicted distinct conductivity values for GM and WM tissues, with notable differences in the range of conductivities observed for specific tissue examples. Despite the variability, many WM and GM tissues exhibited symmetric bilateral conductivities within each microstructure model. SMT yielded conductivity estimates closer to values reported in experimental studies, while none of the methods aligned with spectroscopic models of tissue conductivity.

**Discussion and conclusion:**

Our findings highlight substantial discrepancies in tissue conductivity estimates generated by different diffusion models, underscoring the challenge of selecting an appropriate model for low-frequency electrical conductivity imaging. SMT demonstrated better alignment with experimental results. However other microstructure models may produce better tissue discrimination.

## 1 Introduction

Tissue electrical conductivity properties are closely related to its composition and architecture. Electrical conductivities of different tissues vary widely (over a thousandfold) (1–4) and also depend on temperature and direction. Tissue electrical spectra reflect characteristics of cellular structure and physiological processes and are notably different in disease (5–7). Therefore, conductivity measurements and more importantly, conductivity images, are potentially a valuable diagnostic indication.

Electrical conductivity of isolated tissues is commonly measured by applying a fixed current or voltage across samples via an array of four electrodes (8). The tissue conductivity is then recovered using the relation in Equation 1:

(1) σ=Gκ

where σ is the conductivity in S/m, G is the measured conductance in S, and κ is a shape factor in $m^{-1}$ relating the apparent conductance to the conductivity. This shape factor may be determined independently using the same measurement geometry and a conductivity standard (9).

### 1.1 EIT, MREIT and DT-MREIT

Electrical conductivity imaging has been widely investigated in the area of Electrical Impedance Tomography (EIT) (10). EIT involves application of multiple electrodes to the body surface and obtaining numerous four-electrode conductance measurements. This is then transformed to a conductivity distribution via a sensitivity model connecting apparent conductance to variation of conductivity in each imaged voxel. As the body shape and surface electrode location is difficult to determine accurately, and because this inverse problem is very ill-posed, reconstruction of absolute conductivity distribution is very difficult. The principal cause of EIT’s ill-posedness is the restriction to making boundary measurements. However, magnetic resonance electrical impedance tomography (MREIT) avoids this by using MR coils to measure the magnetic flux created within the body by externally applied currents. Currents are applied synchronously with MRI pulse sequences, images from at least two linearly independent external currents (11) are needed to uniquely reconstruct conductivity distributions. Diffusion tensor MREIT (DT-MREIT) is an extension of MREIT that combines measured magnetic flux data with a subject-specific diffusion tensor to yield conductivity tensor information at the frequency of the applied current (12, 13). MREIT and DT-MREIT have enabled imaging of low-frequency conductivity distributions in the head and knee (14, 15). However, several barriers to clinical adoption exist, most notably in the difficulty of applying external electrodes and the need to adapt sequence timing to accommodate MREIT current administration protocols.

### 1.2 Electric properties tomography

Another MR-based method captures electrical conductivity properties, without electrodes, at the Larmor (resonance) frequency of the MR system. This technique, electric properties tomography (EPT), requires extraction of the phase transmitted by the imaging coil, as this is affected by tissue conductivity and permittivity (16). EPT measurements at higher frequencies (for example in a 3 T MRI system electrical properties are measured at characteristic of 128 MHz) are mostly reflective of tissue composition, as structural information is not apparent in this range. Excellent correspondence has been found between previously obtained conductivity values obtained using coaxial probes (3) and EPT images of brain tissues (17, 18). Intriguing conductivity anomalies have also been observed in tumor tissues (19, 20). However, it is possible that combining structural information from diffusion microstructure images with EPT may improve the specificity of these findings. This approach is the basis of the method we test here, denoted conductivity tensor imaging (CTI).

### 1.3 Conductivity tensor imaging

The recently introduced technique of Conductivity Tensor Imaging (CTI) (21) leverages observations of Basser et al. (22) and Tuch et al. (23) that low-frequency conductivity and diffusion tensors (amongst other physical phenomena) should share common properties. Alternative techniques therefore employ MR diffusion-weighted imaging as a basis for relating diffusion tensors to conductivity tensors.

The relation between conductivity and diffusion tensors is expressed in Equation 2 as

(2) $$C=\eta D^{e}$$

where C is the conductivity tensor, $D^{e}$ is the extracellular diffusion tensor (measured using a low diffusion weighting ca. 800 or 1000 ${s/\mathrm{mm}}^{2}$) and η is an isotropic scaling factor relating conductivity and diffusion properties. In Sajib et al. (21) this factor is derived as in Equation 3:

(3) $$\eta =\frac{\alpha \sigma_{H}}{\alpha d_{e}+\beta \left(1-\alpha \right)d_{i}}.$$

Here, α is the extracellular fraction, $\sigma_{H}$ is the measured EPT conductivity distribution, $d_{e}$ and $d_{i}$ are the extra- and intracellular diffusivity respectively and β is the ratio of intracellular and extracellular ionic concentrations.

The effective isotropic low-frequency conductivity may be calculated using the relation

(4) $$\sigma_{L}=\eta d_{e}=\frac{\sigma_{H}d_{e}\alpha }{\alpha d_{e}+\beta (1-\alpha )d_{i}}$$

Alternatives to CTI exist. For example water-based methods suggested by Michel et al. (24) and Marino et al. (25) relate tissue water content to low-frequency properties, with well-defined results reporting average conductivities in gray and white matter as of 0.55 ± 0.01 S/m and 0.3 ± 0.01 S/m respectively (25).

Both water-based (wEPT), CTI and DT-MREIT values have diverged from model predictions at near DC frequencies (26). In addition, CTI predictions vary as a result of differences in the models used to relate diffusion to conductivity (21, 26–28). One other aspect that has not been explored in CTI or DT-MREIT has been variability in measured gray or white matter conductivities.

In the section below we discuss interpretation of microstructure models in terms of conductivity.

### 1.4 Microstructure diffusion models

As noted in Equation 4 above, low-frequency conductivity properties depend critically on knowledge of intra- and extracellular diffusivity. These can be estimated using diffusion microstructure images, which originally concentrated on characterizing white matter properties. The Neurite Orientation Dispersion and Density Imaging (NODDI) technique, first published in 2012 (29) seeks to highlight white matter orientation patterns. More recently, interest has been focused on other aspects of microstructure composition. For example the Soma and Neurite Density Imaging (SANDI) method (30) emphasizes gray matter model. While SANDI and NODDI make several assumptions of composition or tissue diffusivity, the spherical mean technique (SMT) (31) makes comparatively few and may provide an unbiased tissue diffusivity assessment that may produce conductivities closest to actual values.

### 1.5 Rationale

Depending on the diffusion acquisition used, different CTI studies have predicted a range of low-frequency conductivities for white and gray matter and have not performed detailed analyses of conductivities of specific brain structures. Therefore, the goals of this study were, first, to demonstrate the range of low-frequency conductivity white and gray matter properties predicted using different microstructure models; and second to determine if there are different predicted conductivities within selected brain structures using a microstructure to conductivity transformation. Data from publicly available databases were used. These included volumes from the Human Connectome Project WU-MINN (32) and MGH HCP (33) databases. Conductivities predicted using microstructure information provided by NODDI, SANDI and Spherical Mean (SMT) methods were assessed. We chose to use these three methods because they aim to model white matter, gray matter and general brain tissues respectively and therefore allow us to best determine the likely range of reconstructed conductivities.

In this work we elected to assume high-frequency (EPT) brain tissue conductivities were uniform and individually valued for each tissue type, thus exploring only the effects of diffusion model variations. We anticipate that this study will form the basis for studies to better determine the biophysical basis of LF conductivity properties in brain tissue, and to explore effects of Alzheimer’s disease and cancer on conductivity.

## 2 Methods

### 2.1 Microstructure diffusion models

Currently used clinical standard-of-care diffusion acquisition schemes use a single non-zero diffusion weighting value (for example a b-value of 800 or 1000 ${s/\mathrm{mm}}^{2}$) and assess diffusion along a limited number of diffusion directions (6 or 15) assuming diffusion has a single relaxation characteristic. Advanced diffusion imaging protocols use a wider range of diffusion weightings (more b-values) and directions (b-vectors) to acquire diffusion data. Advanced diffusion imaging methods enable fitting of complex biophysical models to the acquired data. The diffusion imaging models employed here were the Neurite Orientation Dispersion and Density Imaging (NODDI) model, Soma and Neurite Density Imaging (SANDI) model, and the Spherical Mean Technique (SMT). All three biophysical approximations assume Fick’s second law applies i.e., that the location expectation $<x^{2}>$ is proportional to time (34–36). Each model provides quantitative maps of parameters characterizing specific neurite microstructure including as intra- and extra-cellular volumes, and soma and neurite densities and diffusivities. NODDI models specifically distinguish between the extra- and intra-neurite cellular microenvironments by quantifying the diffusion signal obtained from voxels in these regions and modeling them as either a sphere (representing free water or extracellular diffusion), a tensor (representing extra-neurite diffusion) or a stick (representing intra-neurite diffusion). NODDI models are parameterized with quantities including the neurite density index (NDI), orientation dispersion index (ODI), and fractional isotropic volume fraction ($f_{\mathrm{iso}}$). For SANDI, signal and volume fractions are compartmentalized as a zeppelin (representing extracellular space), a sphere (representing intra-soma space) or a stick (representing intra-neurite space). SANDI model parameters include maps of extracellular ($f_{e}$), intra-neurite ($f_{\mathrm{in}}$) and intra-soma ($f_{\mathrm{is}}$) signal fractions and diffusivities. For SMT, metrics are calculated based on averaged extra- and intra-neurite diffusivities and relative volumes. Diffusion metrics for each model were combined to calculate extra- and intracellular diffusivity metrics used in the low-frequency conductivity estimation of Equation 4. Specific conversions used for each method are detailed in the sections below.

### 2.2 Conductivity tensor imaging

We start from the framework defined by Sajib et al. (21). Equation 5 defines the normalized signal arising from each diffusion weighting value b as

(5) $$A_{b}=f_{\mathrm{ecm}}\;e^{-bd_{\mathrm{ecm}}}+f_{\mathrm{ecw}}\;e^{-bd_{\mathrm{ecw}}}+f_{i}\;e^{-bd_{i}}+S_{0}$$

where $f_{\mathrm{ecm}}$ and $d_{\mathrm{ecm}}$ are the volume fraction and diffusivity in the extracellular matrix, $f_{\mathrm{ecw}}$ and $d_{\mathrm{ecw}}$ relate to extracellular water and $f_{i}$ and $d_{i}$ relate to intracellular spaces respectively. $S_{0}$ is a signal offset. Multiple b-values are sampled in the range of 0 to 4,500 ${s/\mathrm{mm}}^{2}$ and each b-value is sampled along multiple directions in order to fit the six parameters $d_{\mathrm{ecm}}$, $d_{\mathrm{ecw}}$, $d_{i}$, $f_{\mathrm{ecm}}$, $f_{\mathrm{ecw}}$ and $f_{i}$. Further, the definition of extracellular fraction (Equation 6) is

(6) $$\alpha =\frac{\;f_{\mathrm{ecm}}+f_{\mathrm{ecw}}}{f_{\mathrm{ecm}}+f_{\mathrm{ecw}}+f_{i}}$$

and extracellular diffusivity (Equation 7) is

(7) $$d_{e}=\frac{\;f_{\mathrm{ecm}}}{f_{\mathrm{ecm}}+f_{\mathrm{ecw}}}d_{\mathrm{ecm}}+\frac{\;f_{\mathrm{ecw}}}{f_{\mathrm{ecm}}+f_{\mathrm{ecw}}}d_{\mathrm{ecw}}$$

It is assumed that $d_{\mathrm{ecw}}=3\times 10^{-3}\;{\mathrm{mm}}^{2}$/s. Fitted or derived parameters for $d_{i}$, $d_{e}$, and α are then used in combination with the reconstructed high-frequency conductivity, via Equation 4 to calculate $\sigma_{L}$.

#### 2.2.1 NODDI

In the NODDI model (29, 37), the normalized signal is described by the relation of Equation 8:

(8) $$A=(1-f_{\mathrm{iso}})(f_{i}A_{\mathrm{ic}}+(1-f_{i})A_{\mathrm{ec}})+f_{\mathrm{iso}}A_{\mathrm{iso}}$$

and multi-b-value diffusion data is fitted to a multicompartment model composed of sticks, isotropic free water space and an anisotropic extracellular matrix. The model has parameters $f_{i}$, the normalized volume fraction of the intracellular compartment, $d_{\|}$, the intrinsic diffusivity in sticks, assumed to be $1.7\times 10^{-3}\;{\mathrm{mm}}^{2}\;s^{-1}$, μ, the mean stick orientation and κ, a concentration parameter that measures the extent of orientation dispersion around the mean stick orientation. The parameters $f_{\mathrm{iso}}$ and $d_{\mathrm{iso}}$ (assumed to be $3.0\times 10^{-3}\;{\mathrm{mm}}^{2}\;s^{-1}$) define the volume fraction and diffusivity of isotropic space, respectively.

The orientation dispersion index (ODI) is a value that varies between 0 and 1 and relates to the parameter κ using (for extracellular space)

(9) $$\kappa =\frac{1}{\tan\;(\pi ODI/2)}$$

In turn, κ relates to the parameter τ, which varies between 1/3 and 1. This is found using Equation 10:

(10) $$\tau =-\frac{1}{2\kappa }+\frac{1}{2F(\sqrt{\kappa })\sqrt{\kappa }}$$

where the function F is defined in Equation 11 as

(11) $$F(x)=\frac{1}{2}\sqrt{\pi }\;e^{-x^{2}}\mathrm{erfi}(x).$$

In this study, NODDI model parameters were translated to effective transverse and parallel intracellular and extracellular matrix diffusivities via the sequence shown in Equation 12:

(12) $$\begin{array}{rl} d_{i∥} & =\nu_{\mathrm{ic}}(1-\nu_{\mathrm{iso}})\times 1.7\times 10^{-3}\;{\mathrm{mm}}^{2}\;s^{-1}\\ d_{i⊥} & =0\\ d_{i} & =\frac{d_{i∥}+2d_{i⊥}}{3}\\ d_{e∥} & =d_{i∥}-d_{i∥}\nu_{\mathrm{ic}}(1-\tau )\\ d_{e⊥} & =d_{i∥}-d_{i∥}\nu_{\mathrm{ic}}\frac{1+\tau }{2}\\ d_{\mathrm{ecm}} & =\frac{d_{e∥}+2d_{e⊥}}{3} \end{array}$$

Finally, conversion to to CTI parameters and conductivities was achieved by computing the extracellular fraction α using the expression of Equation 13 as

(13) $$\alpha =f_{\mathrm{iso}}+\left(1-f_{\mathrm{iso}}\right)\left(1-f_{i}\right)$$

and the total extracellular diffusivity averaged over all directions was calculated using Equation 14 as

(14) $$d_{e}=\frac{\left(1-f_{\mathrm{iso}})(1-f_{\mathrm{ic}}\right)}{f_{\mathrm{iso}}+(1-f_{i})(1-f_{\mathrm{iso}})}d_{\mathrm{ecm}}+\frac{\;f_{\mathrm{iso}}}{f_{\mathrm{iso}}+(1-f_{i})(1-f_{\mathrm{iso}})}d_{\mathrm{iso}}.$$

#### 2.2.2 SANDI

The SANDI model (30, 38) assumes the extracellular space is simpler than for the NODDI model, and places more emphasis on intraneurite (in) and intrasomal (is) spaces. The soma are modelled as closed impermeable spheres and their normalized signal is calculated from Gaussian phase distribution approximations.

The direction-averaged signal for each b-value in the SANDI model is modeled using Equation 15 as

(15) $$A_{b}=-(1-f_{e})\left(\;f_{\mathrm{in}}A_{\mathrm{in},b}+(1-f_{\mathrm{in}})A_{\mathrm{is},b}\right)+f_{e}A_{e,b}$$

where $f_{\mathrm{in}}$ is the relative intraneurite fraction ($f_{\mathrm{in}}+f_{\mathrm{is}}=1$), and $A_{\mathrm{in},b}$ and $A_{\mathrm{is}}$ are the normalized signals for restricted diffusion within neurites and soma respectively. It is assumed that diffusivity in the soma is $D_{\mathrm{is}}=3\;\mu m^{2}/\mathrm{ms}$. Direction-averaged outputs of the SANDI fitting process are $f_{e}$ and $D_{\mathrm{ec}}$ and we estimated SANDI measures to CTI contributions via Equations 16–18:

(16) $$\alpha =f_{e}$$

(17) $$d_{i}=(1-f_{e})(f_{\mathrm{in}}D_{\mathrm{in}}+(1-f_{\mathrm{in}})D_{\mathrm{is}})$$

and

(18) $$d_{e}=f_{\mathrm{ec}}D_{\mathrm{ec}}$$

before conversion to conductivity using Equation 4.

#### 2.2.3 Spherical mean technique

In the spherical mean technique (SMT), (31, 39), diffusion data gathered using at least two b-values are used to estimate the direction-averaged mean diffusion-signal using the model summarized in Equation 19 below as

(19) $$A_{b}=f_{i}A_{i}+\left(1-f_{i}\right)A_{e}$$

The relevant variables solved for by the SMT model are intra-neurite volume fraction ($f_{\mathrm{in}}$), intrinsic diffusivity ($D_{\mathrm{in}}$) and the extra-neurite microscopic mean diffusivity ($D_{e}$).

To convert to CTI parameters we used the associations shown in Equation 20

(20) $$\begin{array}{rl} \;f_{i} & =f_{\mathrm{in}}\\ f_{e} & =1-f_{i}\\ d_{i} & =f_{i}D_{\mathrm{in}} \end{array}$$

followed by calculation of conductivity using Equation 4.

### 2.3 Modeled high-frequency conductivities

**Figure 1:**
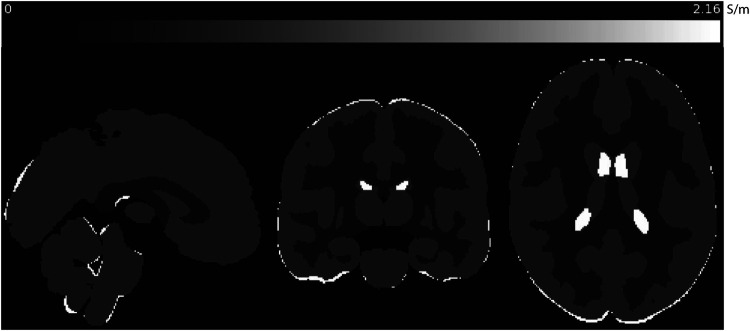
Sagittal, coronal and axial slices of predicted $\sigma_{H}$ values used in this study, shown in MNI space. Conductivities predicted at 128 MHz (3 T) for gray and white matter and CSF were calculated using modeled parameters for each tissue determined by Gabriel et al. (3).

A key factor in Equation 4 is the distribution of high frequency conductivity $\sigma_{H}$. Comprehensive tissue conductivity spectra have been obtained directly using coaxial probes, and empirical models for brain tissue conductivity spectra created by Gabriel et al. (3) have agreed well with previous EPT measurements obtained using MR platforms at 3 and 7 T (17). As the intention of this study was to explore the effect of diffusivity models on predicted low-frequency conductivities, we fixed high-frequency conductivities of white and gray matter and cerebrospinal fluid (CSF) to be those predicted by Gabriel et al.’s models. Briefly, we used atlases of grey and white matter structures (Harvard-Oxford cortical and sub-cortical atlases, and the Johns Hopkins white matter atlas) to assign high-frequency conductivities at 3 T (128 MHz) to each gray matter (GM) and white matter (WM) compartment used in the study, and to cerebrospinal fluid (CSF). At 128 MHz these modeled conductivities were 0.5864 S/m, 0.3420 S/m, 2.1429 S/m respectively. Cross-sectional images of the $\sigma_{H}$ distribution in standard MNI space are illustrated in Figure 1.

### 2.4 Diffusion data sets used

Two data sets were used in the analysis presented in this paper. Both datasets were obtained using 3 T scanners. NODDI and SMT methods were tested using the WU-MINN dataset. The WU-Minn HCP dataset provides diffusion data for 1,200 healthy young adults with b-values (${s/\mathrm{mm}}^{2}$) of 0 (18 directions), 1,000 (90 directions), 2,000 (90 directions) and 3,000 (90 directions). Other settings were TR/TE 5520/89.5 ms; 1.25 mm isotropic voxels. Volumes from the first 199 subjects in the WU-Minn datatset were used to calculate parameters for NODDI and SMT reconstructions. The database used for SANDI fitting was the MGH HCP dataset, which includes diffusion data for 35 adults with b-values (${s/\mathrm{mm}}^{2}$) = 0 (40 directions), 1,000 (64 directions), 3,000 (64 directions), 5,000 (128 directions) and 10,000 (246 directions) with TR/TE 8800/57 ms and 1.5 mm isotropic voxels. We used data from 34 of the 35 subjects in the MGH HCP dataset to calculate SANDI metrics.

### 2.5 Region of interest selection

**Figure 2:**
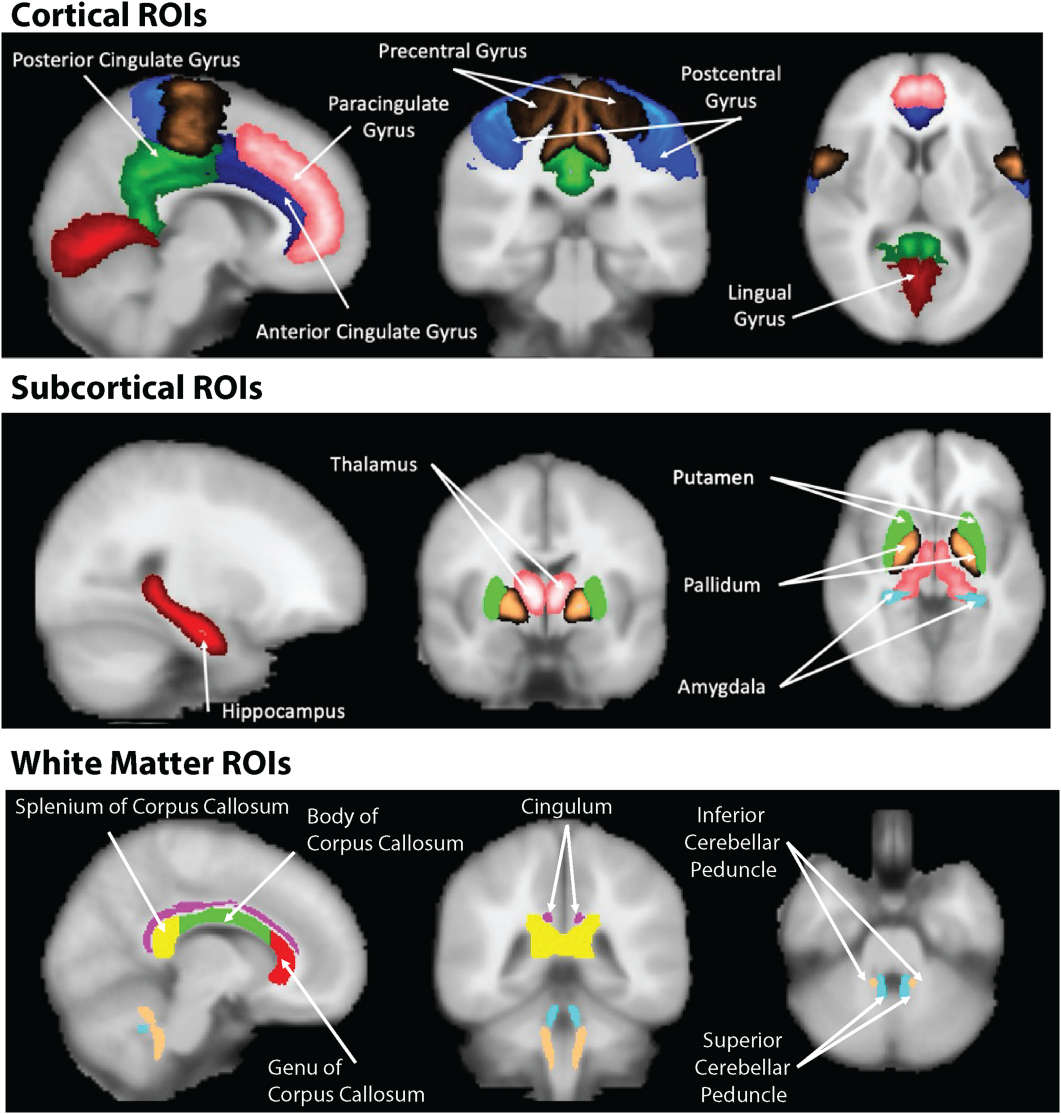
Cortical, subcortical and white matter regions of interest used in the study.

We selected six cortical, five subcortical and six white matter structures for analysis. WM ROIs were selected from the JHU DTI-based white-matter atlases (40–42); cortical and subcortical ROIs were obtained from the Harvard-Oxford cortical and subcortical structural atlases respectively (43–46). WM ROIs chosen were those with large contiguous WM volumes and one compartment in the cerebellum. Subcortical ROIs were chosen to be those likely to include biomarkers of early disease such as the hippocampus, thalamus, amygdala, pallidum and putamen. Cortical ROIs were selected from sensorimotor regions. The cingulate gyrus, and lingual gyri were also assessed. ROIs were further subdivided into left and right, anterior and posterior or superior and inferior sections. As the hippocampus is key to progression of conditions such as Alzheimers and Parkinson’s diseases, we sub-sampled the hippocampus into left and right CA1, CA2 and 3 and CA4DG compartments. ROIs used in the study in each brain region are shown in Figure 2.

### 2.6 Data processing

Parameters for each of the three diffusion methods were determined in each subject’s native space, and resulting volumes for each parameter were transformed into MNI space using a reference 1 mm T1-weighted MNI volume.

NODDI (MATLAB 2024a, The Mathworks, Natick MA, USA) (37) and SMT (39) diffusion models were run on the young adult HCP dataset while SANDI Python code (38) was run on the adult diffusion HCP dataset. All diffusion data was pre-processed incorporating the FSL (FSL fsl.fmrib.ox.ac.uk) eddy function (47) which significantly removes noise caused by subject motion.

Images underwent brain extraction and further processing using the FSL bet tool (48) followed by co-registration of each volume to MNI space using the flirt utility (48). Conductivity measures were calculated using the synthetic high frequency $\sigma_{H}$ maps and diffusion microstructure model parameters using MATLAB. Calculation of region-of-interest (ROI)-specific median and interquartile ranges in CTI maps was performed using fslstats (49).

After co-registration of image volume to MNI space we found that hippocampus or thalamus ROIs of some subjects tended to include CSF. Therefore hippocampus registrations were manually adjusted, and each subject’s thalamus ROIs were eroded to ensure that sample statistics were not contaminated by CSF.

### 2.7 Analyses performed

Statistical testing was performed using R (50). The normality of data for each method and ROI was assessed using the built-in Shapiro-Wilk test (shapiro.test, α<0.05). We found that data in the majority of ROIs were not normally distributed. Therefore non-parametric tests and measures were used to display overall conductivity results using median and interquartile range box and whisker plots. We also used non-parametric tests to determine equivalence of conductivities predicted in corresponding regions within each ROI, for example, left and right hippocampus, and corpus and genu of the corpus callosum. In other instances we compared anterior and posterior parts of the cingulate gyrus and pre- and postcentral gyrus conductivities. Additionally, equivalence tests were performed between each hippocampal subregion. Predicted low frequency CSF conductivity was not explored in this study.

Equivalence analyses were performed using the R TOSTER package (51) (version 0.8.3) using the Wilcoxon signed-rank TOST function wilcox_TOST with a lower equivalence bound at −0.05 and the upper equivalence bound at 0.05 and α<0.05. The wilcox_TOST function calculates the median difference between groups, the pooled standard deviation, and the standard error, and determines if this difference falls within equivalence bounds. It also provides a two-sided test of equivalence, generating confidence intervals and p-values to evaluate if the observed differences are statistically significant or fall within the range of equivalence. This non-parametric test was chosen because it does not assume normality and is appropriate for comparing two independent groups.

## 3 Results

### 3.1 Overall comparisons

**Figure 3:**
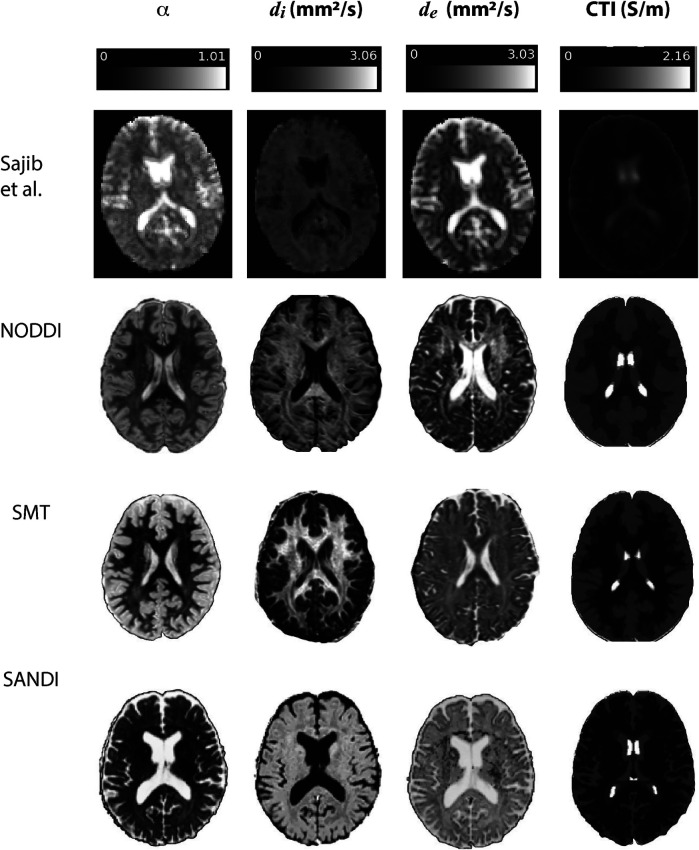
Comparison of results found in Sajib et al. (26) in a single subject against example α, $d_{i}$ and $d_{e}$ quantities extracted for individual subjects in NODDI, SMT and SANDI analyses.

Examples of contributing parameters and low-frequency conductivities predicted by each method are displayed in Figure 3 for individual subjects. Also shown in this figure is an example of parameters and low frequency conductivity found in vivo by Sajib et al. (26). Note that the data slice and subject used to determine the example NODDI, SMT and SANDI parameters was the same slice of MNI space in each case, while that shown in the Sajib. et al data was estimated over a different, 5 mm slice. Differences between parameters estimated by each method were clearly evident, with α values estimated in both gray and white matter being larger in NODDI and SMT methods than in SANDI, and $d_{i}$ appearing larger in SANDI than for other methods. Overall, gray and white matter conductivities predicted by the NODDI method were larger than those predicted with SANDI, with SMT predictions sitting between these two.

### 3.2 ROI conductivity distributions

**Figure 4:**
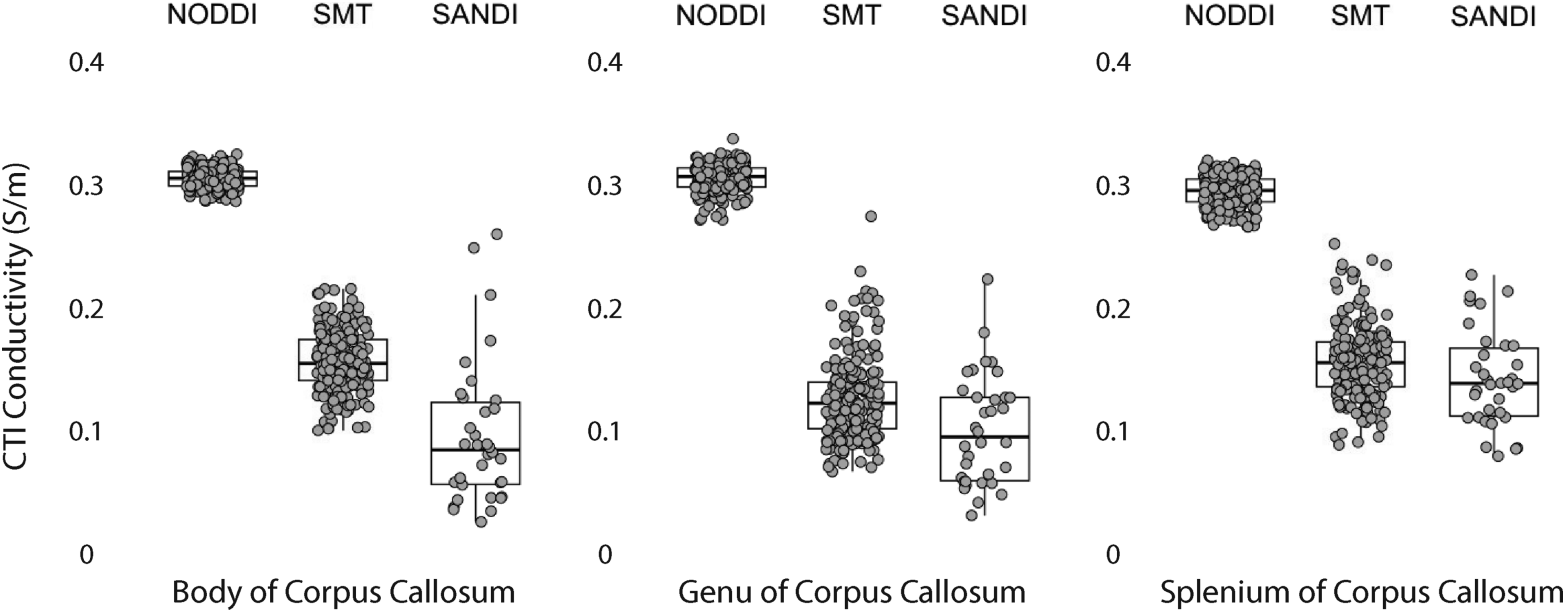
CTI conductivities predicted in corpus callosum subcompartments by by NODDI, SMT and SANDI methods. Each filled circle indicates median value of conductivity in each structure for each method. Box and whisker plots show the median, interquartile range (box), and the range excluding outliers (whiskers). Identical data (N = 199, WU-MINN) was used for NODDI-CTI and SMT-CTI images. Data for SANDI-CTI reconstructions were obtained from the MGH-HCP database (N = 34).

**Figure 5:**
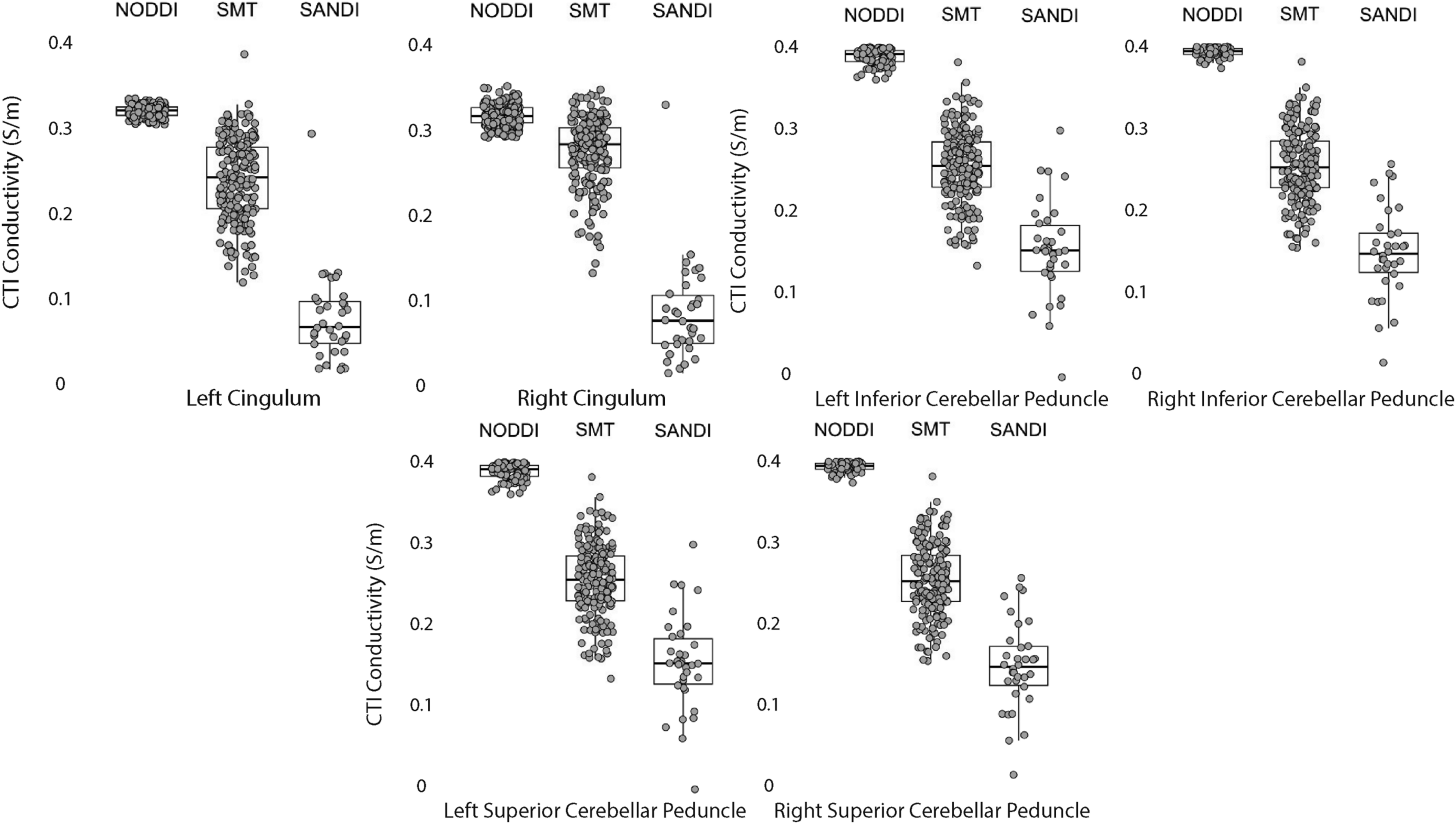
CTI conductivities predicted in white matter ROIs by NODDI, SMT and SANDI methods. Each filled circle indicates median value of conductivity in each structure for each method. Box and whisker plots show the median, interquartile range (box), and the range excluding outliers (whiskers). Identical data (N = 199, WU-MINN) was used for NODDI-CTI and SMT-CTI images. Data for SANDI-CTI reconstructions were obtained from the MGH-HCP database (N = 34).

**Figure 6:**
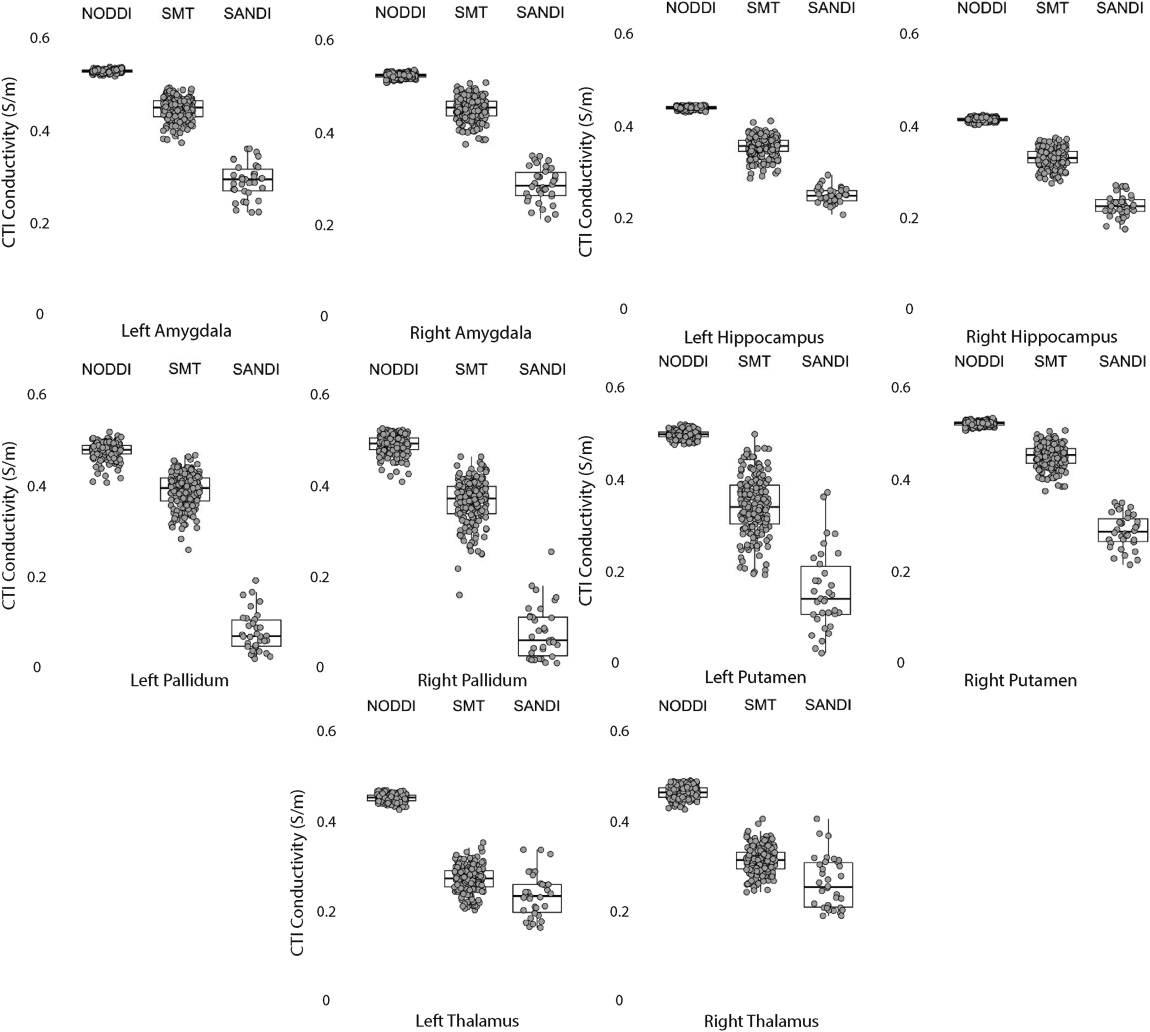
CTI conductivities predicted in subcortical ROIs by NODDI, SMT and SANDI methods. Each filled circle indicates median value of conductivity in each structure for each method. Box and whisker plots show the median, interquartile range (box), and the range excluding outliers (whiskers). Identical data (N = 199, WU-MINN) was used for NODDI-CTI and SMT-CTI images. Data for SANDI-CTI reconstructions were obtained from the MGH-HCP database (N = 34).

**Figure 7:**
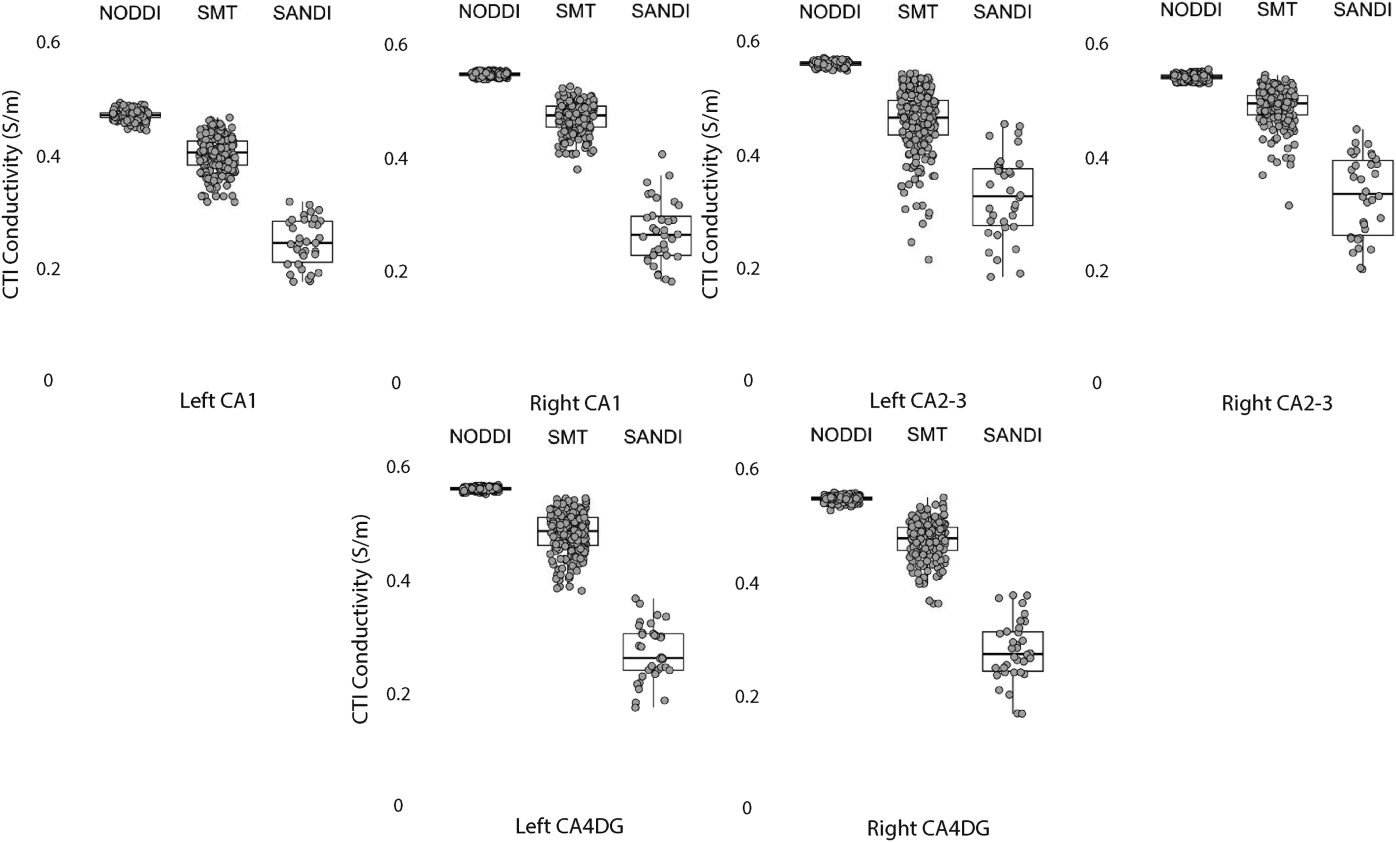
CTI conductivities predicted in left and right hippocampus ROIs found by each method. Each filled circle indicates median value of conductivity in each structure for each method. Box and whisker plots show the median, interquartile range (box), and the range excluding outliers (whiskers). Identical data (N = 199, WU-MINN) was used for NODDI-CTI and SMT-CTI images. Data for SANDI-CTI reconstructions were obtained from the MGH-HCP database (N = 34).

**Figure 8:**
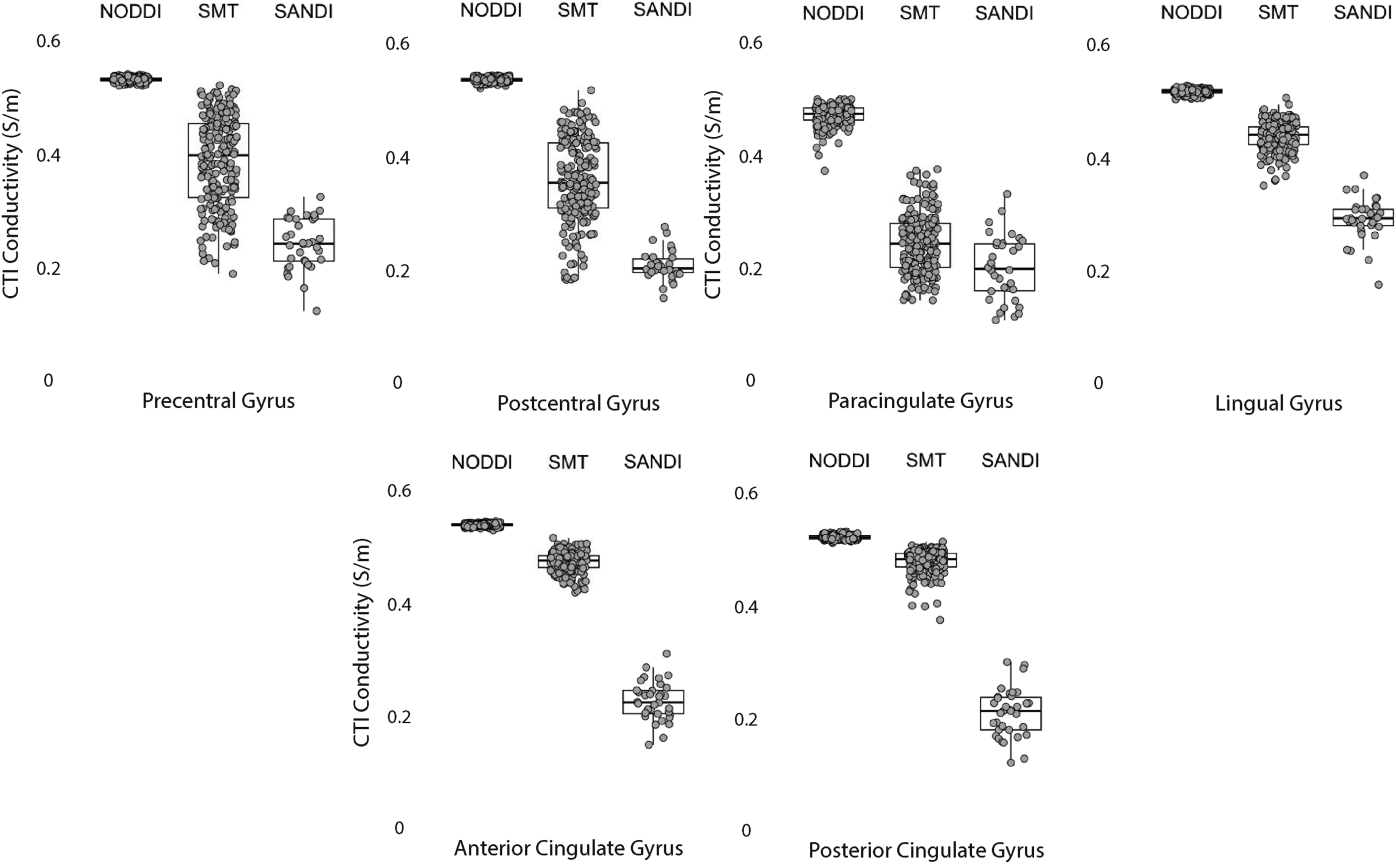
CTI conductivities predicted in cortical ROIs by NODDI, SMT and SANDI methods. Each filled circle indicates median value of conductivity in each structure for each method. Box and whisker plots show the median, interquartile range (box), and the range excluding outliers (whiskers). Identical data (N = 199, WU-MINN) was used for NODDI-CTI and SMT-CTI images. Data for SANDI-CTI reconstructions were obtained from the MGH-HCP database (N = 34).

Data within each ROI class are summarized in box and whisker plots showing the median ± interquartile ranges in conductivity within each class of ROI in Figures 4–8. Marker circles indicate medians of each subject from either 199 subject sampled from the WU-Minn HCP database (NODDI and SMT) or the 34 subjects used from the MGH HPC database (SANDI). Specifically, predicted conductivities in genu, body and splenium segments of the corpus callosum are summarized in Figure 4; those in other white matter ROIS (cingulate gyrus, cerebellar peduncle) are shown in Figure 5. Those for key subcortical ROIs including amygdala, hippocampus, pallidum, putamen and thalamus are to be found in Figure 6 and cortical ROI results are shown in Figure 8.

### 3.3 Equivalence findings

**Figure 9:**
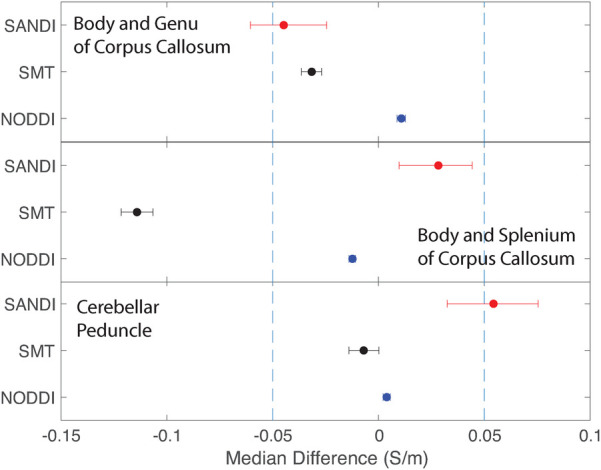
Equivalence test results evaluating congruence of CTI conductivities found between comparable white matter ROIs for each microstructure fitting method. (Top) Body and Genu and (middle) Body and Splenium of Corpus Callosum, and (bottom) inferior superior cerebellar peduncle.

**Figure 10:**
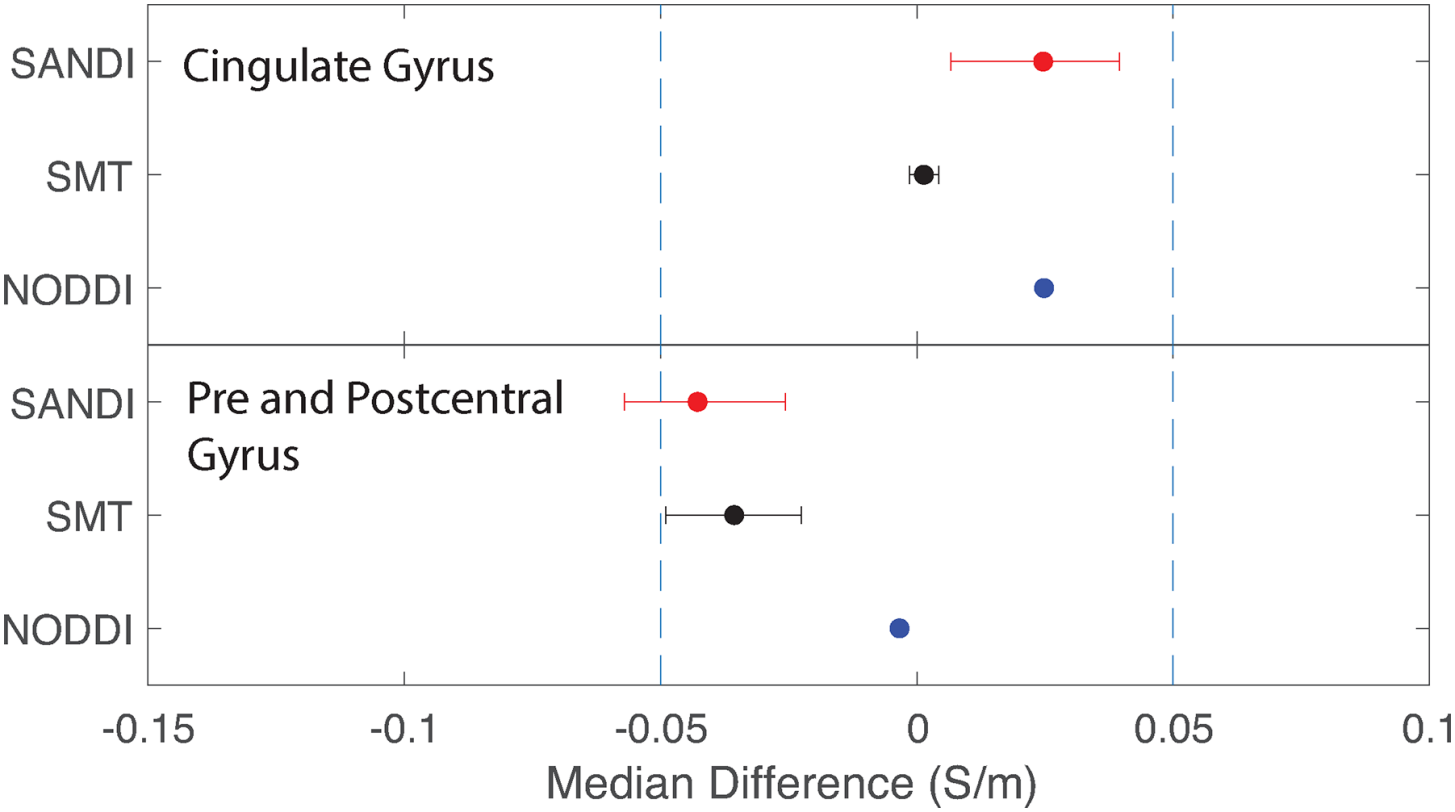
Equivalence test results evaluating congruence of CTI conductivities found between comparable cortical ROIs for each fitting method. (Top) anterior and posterior cingulate gyrus (bottom) pre and postcentral gyrus.

**Figure 11:**
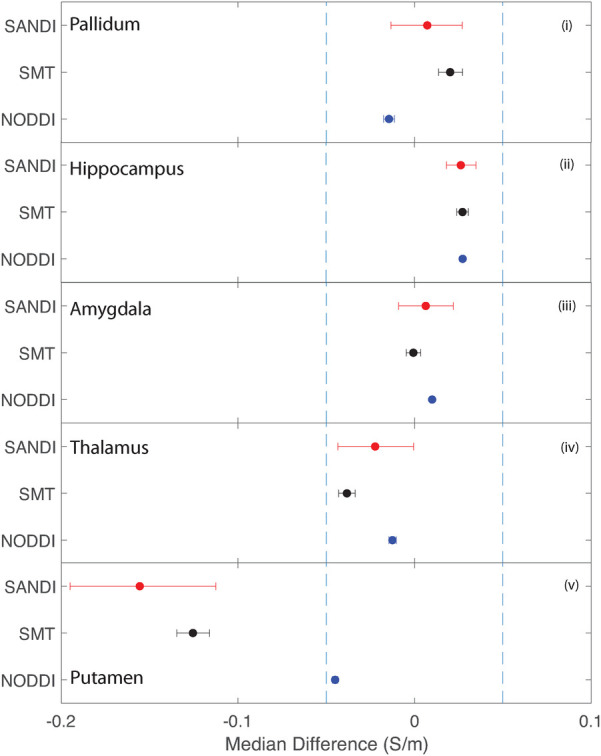
Equivalence test results evaluating congruence of CTI conductivities found between comparable subcortical ROIs for each fitting method. (**i**) Left and right pallidum (**ii**) left and right hippocampus, (**iii**) left and right amygdala, (**iv**) left and right thalamus and (**v**) left and right putamen.

**Figure 12:**
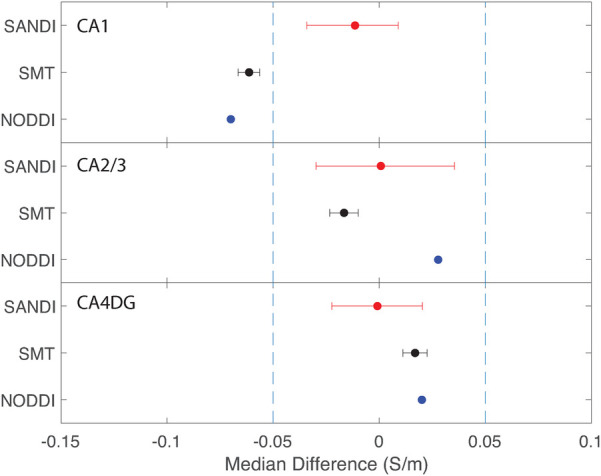
Equivalence test results evaluating congruence of CTI conductivities found between left and right hippocampus subfields. (Top) left and right CA1, (middle) left and right CA2 and 3, (bottom) left and right CA4DG.

Equivalence findings for comparable pairs of ROIs or ROI subcompartments are summarized in Figures 9–12. The specified equivalence bounds of −0.05 and +0.05 S/m are indicated in each plot as dashed lines.

Conductivities predicted in white matter compartments were compared between body and genu of corpus callosum, body and splenium of corpus callosum and inferior and superior cerebellar peduncle. For each pair of white matter ROIs, comparisons for each method were statistically different (median difference non-zero). Predictions for SMT and NODDI models were equivalent in body and genu of corpus callosum; but SANDI comparisons were not equivalent between these ROIs. Comparisons for SANDI and NODDI models were equivalent for body in splenium of corpus callosum, but not equivalent for SMT. For left and right superior cerebellar peduncle, predicted SMT and NODDI conductivities were equivalent, but SANDI predictions were not equivalent.

Median conductivities between cortical ROIs were all significantly different. Anterior and posterior cingulate gyrus conductivities were predicted to be equivalent for each diffusion model. While pre and postcentral gyrus conductivties were predicted by equivalent by NODDI models, and marginally by SMT models, those predicted by SANDI models were not equivalent.

Conductivities predicted in subcortical ROIs were all different, with the exception of left and right amygdala, where predicted conductivities in left and right amygdala were found to be statistically similar. All predicted conductivities for left and right pallidum, hippocampus, amygdala and thalamus were equivalent for each diffusion model. Non-equivalent conductivities were observed between left and right putamen ROIs for SANDI and SMT models. Predicted conductivities in left and right putamen were equivalent for NODDI models only.

Comparisons between left and right hippocampal structures found statistically similar conductivities predicted in left and right CA1, CA2/3 and CA4DG ROIs for SANDI diffusion models. Comparisons for the NODDI and SMT models were all statistically different, and equivalence was observed for all methods and ROIS except for the case of CA1, where both left and right SMT and NODDI predictions were not equivalent.

## 4 Discussion

In the sections below we discuss overall findings and their implications for further investigations linking conductivity and diffusion parameters.

### 4.1 Differences between models

The most prominent observation in the study is the narrow distributions predicted by NODDI models compared with SMT and SANDI predictions. As noted above, NODDI models focus on identification of white matter, and some properties such as the intrinsic stick diffusivity and diffusivity in isotropic spaces are assumed. Therefore, we found that overall predicted NODDI conductivities were dominated by these assumptions, and led to much narrower predicted conductivity ranges in each ROI. By contrast, variance in SMT model predictions (derived from the same data used for NODDI predictions) was much larger, and conductivities smaller than those found for NODDI models. In addition, median NODDI conductivity predictions did not differ greatly between gray or white matter. Predicted conductivities for SANDI models were somewhat more variable than for NODDI cases. SANDI models assume a single parameter (the diffusivity in the soma). Median predictions for SANDI were also somewhat lower than those found for NODDI or SMT models, although we note that SANDI model data were not directly comparable as they were measured using different subjects and sequences from those analyzed in NODDI and SMT models.

### 4.2 Predicted gray and white matter conductivities

White and gray matter estimations found by low-frequency MRI-based measurements have consistently been larger than those anticipated by Gabriel’s parametric models of <0.1 S/m (3, 26). Conductivity measurements derived from water-based measurement (25), CTI (27, 28) or MREIT strategies (14) have found average white matter conductivities ranging from 0.2 S/m to 0.3 S/m, and those of gray matter to be average in the range 0.3 S/m–0.5 S/m.

**Figure 13:**
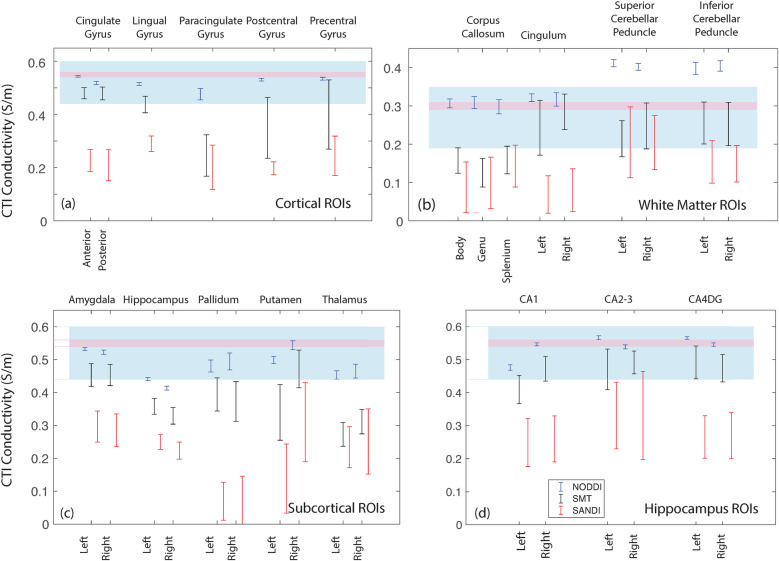
Comparison of the median WM and GM predictions from each method and ROI with values determined by Jahng et al. (27) and Marino et al. (25). **(a)** values for cortical ROIs, **(b)** white matter ROIs, **(c)** subcortical ROIs and **(d)** hippocampal ROIs.

Figure 13 overlays average white and gray matter conductivities reported by Jahng et al. (27) and Marino et al. (25) over median NODDI, SMT and SANDI predictions in each ROI category. Figure 13 overlays a range one standard deviation either side of the mean gray matter conductivity found by Jahng et al. (27) (in blue) over (Figure 13a) cortical, (Figure 13c) subcortical and (Figure 13d) hippocampal subfield ROIs and, similarly, gray matter conductivities found by Marino et al. (25) are overlaid on these parts of the figure in pink. Figure 13b overlays white matter averages found for white matter tissues in these studies over white matter ROI findings for the study.

For ROIs consisting of only white matter, such as the body, genu and splenium of the corpus callosum, conductivities predicted by SMT and SANDI models were between 0.1 S/m and 0.15 S/m Figure 4, lower than averages found in previous studies (14, 27). The most direct comparison of our predictions to external work was of average SMT-derived conductivities found by Jahng et al. (27). White matter conductivity values found in (27) averaged around 0.27 S/m, lower than those found in corpus callosum. However, predicted SMT-derived conductivities in other white matter structures (cingulum, cerebellar peduncle) agreed better, averaging about 0.25 S/m.

In the subcortical and cortical structures, conductivities predicted by all models were overall higher, as expected. Median NODDI conductivities varied between 0.45 S/m and 0.59 S/m, while SMT medians ranged from around 0.2 S/m to about 0.35 S/m. SMT median conductivities in gray matter were in the range 0.25 S/m to 0.5 S/m. SMT means reported in (27) were about 0.52 S/m, slightly above the range found here. Overall predicted conductivities of cortical and subcortical structures tended to lie within with the range of values found in previous studies with some notable exceptions in the case of SANDI predictions and some SMT-derived values including the hippocampus, pallidum and putamen.

### 4.3 Differences between structures

Comparisons of different white matter structures found that purely white matter ROIs (subcompartments of the corpus callosum) had lower conductivities than other white matter structures, possibly reflecting differences in white matter fiber density and radius (52), and possibly also cell composition. In subcortical gray matter compartments we also found varying conductivity for each compartment, likely related to fractional volume effects of white matter tracts traversing each structure. For example the conductivity predicted in the pallidum, a structure that has a large number of white matter tracts was particularly low for the SMT and SANDI models, which may be a result of white matter content. Conductivities predicted by both SMT and SANDI models for the hippocampus, thalamus and left putamen also had distinctly low values. Within the hippocampal subcompartments, there were differences noted both in distribution and median conductivities, between CA1 against CA2-3 and CA4. Mixing gray and white matter tissue may also be the cause of the lower and variable conductivities predicted by SMT and SANDI models for the pre- and post-central gyrus and paracingulate gyrus.

### 4.4 Bilateral or anterior-posterior comparisons

We also tested correspondence of conductivities predicted by each model for the different ROIs, either by comparing left with right, anterior with posterior or inferior and superior sub-structures. In most cases there was equivalence found between models for these symmetry tests. However, notable discrepancies were observed in the putamen and CA1 subcompartment of the hippocampus.

### 4.5 Study limitations

The main limitation of this study was the lack of knowledge of the high frequency conductivity contribution to predicted conductivities $\sigma_{H}$. However, this provides a platform against which variations in diffusion parameters alone can be evaluated in conductivity terms.

A second limitation is that fixed imaging parameters have been used to determine the diffusion characteristics, and these may not be the optimal ones to determine conductivity characteristics. In future studies, it would be advisable to determine the effect of varying the diffusion b values or diffusion times on reconstructed parameters.

We also recognize that the NODDI methods employed in this study do not capture microstructural details related to the soma (gray matter) or cellular exchange across tissue microenvironments, both of which are crucial aspects of microstructure. Also, while NODDI is traditionally associated with white matter, our study extends its application to gray matter regions, such as the hippocampus. Although these models may not fully capture the complexity of gray matter microstructure or cellular exchange, our findings suggest that they still provide valuable insights into the microstructural environment of these regions. By carefully considering the limitations of these models in gray matter, our study demonstrates the potential of integrating diffusion-based metrics with conductivity measurements to enhance our understanding of tissue microstructure in both white and gray matter. This approach holds promise for future applications in sensitive monitoring of structural and compositional changes across different brain regions. The main limitations of NODDI include the lack of direct diffusivity estimation and the potential bias in its parameters due to fixed diffusivities (53). Additionally, while NODDI has been shown to have good repeatability and reproducibility, its application in clinical studies must account for intra- and inter-subject variability (54). Another significant limitation is the spatial resolution of diffusion-weighted imaging, which can be problematic in small or complex brain regions like the hippocampal subfields, leading to partial volume effects where signals from different tissue types are mixed, potentially confounding the results (55).

Raw data was made available to us through the databases and in already preprocessed form (i.e., corrected for motion and susceptibility artifact). However due to multiple inherent factors, some artifacts such as ghosting artifact were still apparent especially in the case of the SANDI data as scanning at higher b-values and for exceedingly long scan times can contribute to patient motion and EPI (echo-planar-imaging) distortions.

Finally, data assessments in this study were made after transformation of conductivity tensor volumes to MNI space. Careful efforts were made to ensure that ROIs were not contaminated by adjacent tissues, but it could be the case that some observations have been affected by this factor, particularly in the putamen and hippocampus.

### 4.6 Future directions

Another issue that might be of interest is that of sex differences, which have not been explored here. We plan to include this analysis in a wider exploration of the HCP 1200-subject database. Differences observed in the hippocampus may also be the basis for distinguishing disease. Initial predictions using NODDI metrics and diffusion data collected on carriers of APOE-ϵ4 gene markers associated with Alzheimer’s disease have indicated that conductivities predicted in hippocampal sub-compartments are statistically different from those observed in age-matched controls, and this will be a focus of future studies.

### 4.7 Overall findings

We have elected to keep these preliminary findings qualitative pending further validation of conductivity tensor imaging methods against structure and cell morphology, and other imaging methods. Overall, conductivity predictions in ROIs broadly reflected the mix of tissue types within each structure, which shows promise that CTI will find multiple applications in sensitive monitoring of small variations in tissue structure and composition.

We found that SANDI and NODDI predictions had less variance than SMT predictions, which is likely the result of assumptions made in SANDI and NODDI models. While SANDI and NODDI results may not produce accurate conductivity data, we speculate the combination of components used to produce NODDI- or SANDI-CTI values may yield useful and sensitive biomarkers for tissue state that may improve disease prediction.

As high frequency conductivity data was not used here, we anticipate that its inclusion could potentially amplify sensitivity to small tissue changes, particularly in ionic composition. However, inclusion of real EPT data could also increase variability in reconstructed conductivities, obscuring the sensitivity provided by diffusivity metrics.

This study suggests that low-frequency conductivity imaging metrics could serve as a sensitive biomarker for tissue structure and composition. Since many diffusion and conductivity metrics change with pathology, CTI measures are likely similarly affected and the method has promise for detection of early stage neurological diseases or cancers.

## Data Availability

Publicly available datasets were analyzed in this study. This data can be found here: Human Connectome Project Young Adult Data Release https://www.humanconnectome.org/study/hcp-young-adult/document/1200-subjects-data-release Human Connectome Project MGH-HCP Data Release https://humanconnectome.org/study/hcp-young-adult/document/mgh-adult-diffusion-data-acquisition-details.
